# Multi-Harmonic Modulation in a Fiber-Optic Gyroscope

**DOI:** 10.3390/s23094442

**Published:** 2023-05-01

**Authors:** Martin Miranda, Nobuyuki Takei, Yuki Miyazawa, Mikio Kozuma

**Affiliations:** 1 Institute of Innovative Research, Tokyo Institute of Technology, 4259 Nagatsuta-cho, Midori-ku, Yokohama, Kanagawa 226-8503, Japan; miranda@qnav.iir.titech.ac.jp (M.M.); takei@qnav.iir.titech.ac.jp (N.T.); miyazawa@qnav.iir.titech.ac.jp (Y.M.); 2 MIZUSAQI Inc., 1-48-3 Hanasaki-cho, Naka-ku, Yokohama, Kanagawa 231-0063, Japan; 3 Department of Physics, Tokyo Institute of Technology, 2-12-1 O-Okayama, Meguro-ku, Tokyo 152-8550, Japan

**Keywords:** fiber-optic gyroscope, multiple harmonics, sinusoidal bias modulation, relative-intensity noise, thermal-phase noise

## Abstract

Optimizing the bias modulation of a fiber-optic gyroscope is crucial to improving its precision. In this study, we propose and demonstrate the use of multiple harmonics of sinusoidal modulation as an intermediate alternative to the widely used modulation methods: sinusoidal and square-wave modulation. We show that this alternative integrates the advantages of each modulation method by providing a smooth modulation that produces a clean, spike-free output and a satisfactory signal-to-noise ratio. By using three harmonics of modulation in combination with a high frequency to reduce thermal phase noise, we obtained an angular random walk of $5.2\left(2\right)\;\mu \deg/\sqrt{h}$ and a bias instability of ∼10μdeg/h. This is the highest performance ever reported for fiber-optic gyroscopes.

## 1. Introduction

Inertial rotational motion detection is an essential requirement in the fields of inertial navigation and geophysics. Based on the Sagnac effect, interferometric fiber-optic gyroscopes (FOGs) measure the phase difference of two counterpropagating light waves traveling through a fiber coil. In recent years, FOGs have received extensive attention and research [1,2,3] because of their light weight, high reliability, wide dynamic range, and long life.

For demanding applications, including inertial navigation and geophysics, improving the precision of an FOG is crucial. The precision of an FOG can be improved by either increasing its scale factor, which is proportional to the coil diameter and length, or reducing its amount of phase noise [4]. Giant FOGs with large coil diameters of over a meter are promising for studying geophysical phenomena [4,5]. Using longer fibers is also possible at the expense of increased shot noise contributions due to optical attenuation. While considering the effects of shot noise, the optimal coil length is a few kilometers [6].

Reducing phase noise is especially important in applications regarding inertial navigation because the precision can be improved without increasing the size of the coil [4,7]. In an FOG interrogated by incoherent light, four types of noise dominate [8]: relative-intensity noise (RIN) [9,10,11,12], thermal-phase noise (TPN) [13,14,15], shot noise (SN), and detection noise (DN). While SN and DN can be kept sufficiently small using high-power laser sources and low-noise detection instruments, ultimately, RIN and TPN are the limiting factors in current FOGs. To reduce TPN, high modulation frequencies can be used. This has been demonstrated in previous experiments that are based on using a 30km-long single-mode (SM) fiber coil [16]. Several techniques were demonstrated to reduce the effects of RIN: using noise subtraction by either electronic [17] or optical [18,19,20] means; with an interferometric filter [21]; with a fiber ring resonator [22]; using a semiconductor amplifier [23]; and by current feedback [24].

To properly identify the rotation direction, an FOG utilizes dynamic biasing phase modulation. Optimization of the bias modulation is essential for reducing the phase noise [25]. Two different types of modulation are used on FOGs: sinusoidal and square-wave modulation (SWM). Sinusoidal modulation is popular in open-loop-based FOG since it can be easily achieved with commercial lock-in amplifiers. On the other hand, in terms of maximizing the ratio between the sensitivity and RIN, SWM is preferred [25]. This is because the signal is proportional to sin(ϕ), where ϕ is the modulation amplitude, while RIN is proportional to 1+cos(ϕ). Regarding a modulation depth near ϕ=π, both the signal and RIN converge to zero, but the signal-to-noise ratio diverges to infinity [25].

Unfortunately, using SWM has disadvantages. When the modulation voltage switches at a finite speed, spikes are generated in the detected signal. Spikes can be partially removed from the signal by analog or optical switching [25] or digital processing. However, since the temporal shape of the spike is determined only by the bandwidth of the modulation and detection elements and is independent of the frequency, the temporal density of spikes increases with frequency. A large portion of the signal must be removed at high modulation frequencies, which deteriorates the sensitivity. Therefore, the following dilemma arises: choosing a high modulation frequency that favors TPN reduction or using SWM, which reduces RIN but is incompatible with high modulation frequencies due to the spike problem.

In this paper, we propose the use of multi-harmonic modulation for solving this dilemma. A modulation signal with multiple harmonics is given by:

(1) $$\phi_{\mathrm{mod}}=\sum_{n=1}^{N_{h}}\phi_{2n-1}\sin\left(2n-1\right)\omega t$$

where ω and $\phi_{2n-1}$ are the odd-order harmonic of the fiber-coil eigenfrequency and the modulation depth, respectively. This includes the sinusoidal wave modulation case for $N_{h}=1$ and the square-wave modulation for $N_{h}\to \infty$, given by:

(2) $$\phi_{\mathrm{sq}}=\frac{4\phi }{\pi }\sum_{n=1}^{\infty }\frac{1}{2n-1}\sin\left(2n-1\right)\omega t.$$

where ϕ is the amplitude of the square wave. We show that choosing a multi-harmonic bias modulation that is sufficiently close to a square wave modulation can reduce the RIN effects. Additionally, since this modulation does not generate spikes, it can be applied at high frequencies to reduce TPN.

The goals of this work are to derive theoretical formulas for multi-harmonic modulation, obtain optimal parameters for modulation, and experimentally verify its potential. In particular, we focus on the cases for $N_{h}=1$, 2 and 3 corresponding to single-harmonic modulation (SHM), dual-harmonic modulation (DHM) and triple-harmonic modulation (THM), respectively.

## 2. Theory of Multi-Harmonic Modulation

First, SHM will be reviewed. The output of the photodetector is given by:

(3) $$S=\frac{I_{0}}{2}ℜ\left(1+e^{i\theta }e^{i\phi_{1}\sin\omega t}\right),$$

where $I_{0}$ is the current at the photodetector without rotation and modulation and θ is the Sagnac phase. The Jacobi-Anger expansion can be used to obtain:

(4) $$e^{i\phi_{1}\sin\omega t}=\sum_{n=-\infty }^{\infty }J_{n}\left(\phi_{1}\right)e^{in\omega t}$$

where $J_{n}\left(\phi_{1}\right)$ is the *n*-th order ordinary Bessel function (OBF) of the first kind. Moreover, we obtain:

(5) $$S=\frac{I_{0}}{2}ℜ\left(1+e^{i\theta }\sum_{n=-\infty }^{\infty }J_{n}\left(\phi_{1}\right)e^{in\omega t}\right).$$

The demodulated, fundamental harmonic of the signal results in:

(6) $$S_{1\omega }=\int_{-\infty }^{\infty }S\sin\left(\omega t\right)dt=-I_{0}\sin\theta J_{1}\left(\phi_{1}\right).$$

Similarly, the output of the photodetector modulated by DHM is:

(7) $$S=\frac{I_{0}}{2}ℜ\left(1+e^{i\theta }e^{i\phi_{1}\sin\omega t+i\phi_{3}\sin3\omega t}\right).$$

We can now introduce the two-dimensional generalized Bessel function (GBF) [26,27], with a Jacobi-Anger expansion given by:

(8) $$e^{i(u\sin p\omega t+v\sin q\omega t)}=\sum_{n=-\infty }^{\infty }J_{n}^{p,q}\left(u,v\right)e^{in\omega t}$$

Then, the photodetector signal results in:

(9) $$S=\frac{I_{0}}{2}ℜ\left(1+e^{i\theta }\sum_{n=-\infty }^{\infty }J_{n}^{1,3}\left(\phi_{1},\phi_{3}\right)e^{in\omega t}\right).$$

Since this equation is in the same form as Equation (5) except for a different factor multiplying $e^{in\omega t}$, it follows that:

(10) $$S_{1\omega }=-I_{0}\sin\theta J_{1}^{1,3}\left(\phi_{1},\phi_{3}\right).$$

The two-dimensional GBF can be decomposed into ordinary Bessel functions:

(11) $$J_{n}^{p,q}\left(u,v\right)=\sum_{k,l\in Z,\;pk+ql=n}J_{k}\left(u\right)J_{l}\left(v\right)$$

For $\phi_{3}=0$, the following can be obtained:

(12) $$J_{n}^{1,3}\left(\phi_{1},0\right)=\sum_{k,l\in Z,\;k+3l=n}J_{k}\left(\phi_{1}\right)J_{l}\left(0\right)=J_{n}\left(\phi_{1}\right),$$

and Equation (10) reverts to Equation (6), that is, the SHM case.

As seen with Equations (5) and (9), any Jacobi-Anger expansion in the SHM case can be expressed in an equivalent form for the DHM case by just replacing the OBF with the GBF. Using this property, known equations for RIN and SN [24] can be easily extended to the DHM case to obtain:

(13a) $$\sigma_{\mathrm{RIN}}=\frac{\eta I_{0}\sqrt{B}}{2\sqrt{\Delta \nu }}{\left\{{\left[1+J_{0}^{1,3}\left(\phi_{1},\phi_{3}\right)-J_{2}^{1,3}\left(\phi_{1},\phi_{3}\right)\right]}^{2}+\sum_{n=1}^{\infty }{\left[J_{2n}^{1,3}\left(\phi_{1},\phi_{3}\right)-J_{2n+2}^{1,3}\left(\phi_{1},\phi_{3}\right)\right]}^{2}\right\}}^{1/2}$$

(13b) $$\sigma_{\mathrm{SN}}={\left\{\frac{eI_{0}B}{2}\left[1+J_{0}^{1,3}\left(\phi_{1},\phi_{3}\right)-J_{2}^{1,3}\left(\phi_{1},\phi_{3}\right)\right]\right\}}^{1/2}$$

where *B*, Δν, η and *e* are the detection bandwidth, laser spectrum bandwidth, attenuation factor related to semiconductor gain effects, and electron charge, respectively.

Additionally, this analysis can be further extended to the THM case using the three-dimensional GBF [27] given by:

(14a) $$e^{i(u\sin p\omega t+v\sin q\omega t+w\sin r\omega t)}=\sum_{n=-\infty }^{\infty }J_{n}^{p,q,r}\left(u,v,w\right)e^{in\omega t}$$

(14b) $$J_{n}^{p,q,r}\left(u,v,w\right)=\sum_{k,l,m\in Z,\;pk+ql+rm=n}J_{k}\left(u\right)J_{l}\left(v\right)J_{m}\left(w\right).$$

The equations for an increased number of harmonics $N_{h}>3$ are straightforward.

## 3. Modulation Index Optimization

The theoretical formulas for the signal and each of the noises can be used to obtain the optimal set of modulation depths $\left(\phi_{1},\phi_{3},\phi_{5}\right)$ that minimize RIN- or SN-induced angular random walk (ARW); this set is defined by:

(15a) $${\mathrm{ARW}}_{\mathrm{RIN}}\left(\phi_{1},\phi_{3},\phi_{5}\right)=\frac{\sigma_{\mathrm{RIN}}}{S_{F}I_{0}J_{1}^{1,3,5}\left(\phi_{1},\phi_{3},\phi_{5}\right)\sqrt{B}}$$

(15b) $${\mathrm{ARW}}_{\mathrm{SN}}(\phi_{1},\phi_{3},\phi_{5})=\frac{\sigma_{\mathrm{SN}}}{S_{F}I_{0}J_{1}^{1,3,5}\left(\phi_{1},\phi_{3},\phi_{5}\right)\sqrt{B}}$$

respectively [24]. Here, $S_{F}=4\pi RL/c\lambda$ is the scale factor of a fiber coil with a radius of *R* and a length of *L*. Moreover, *c* and λ correspond to the speed of light and central wavelength of the light source, respectively. To perform the analysis, it is convenient to plot ARW values as a function of the modulation index $\phi_{1}$ and the ratios ($\phi_{3}/\phi_{1}$, $\phi_{5}/\phi_{1}$), which determine the depth and temporal shape of the modulation signal, respectively.

**Figure 1:**
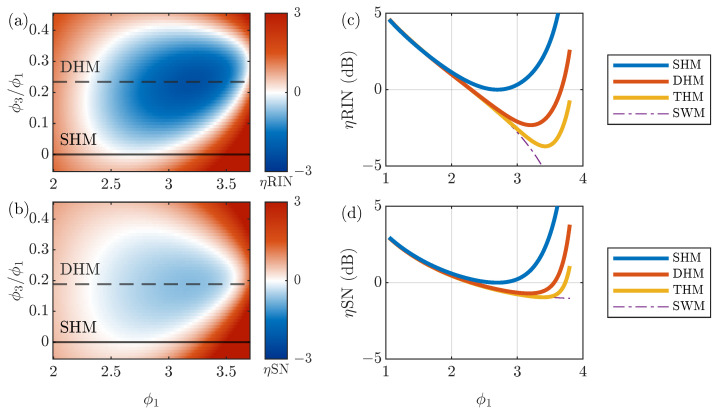
Improvement of RIN- and SN-induced ARW for multi-harmonic modulation. Improvement units are in dB. (**a**,**b**): $\eta_{\mathrm{RIN}}$ and $\eta_{\mathrm{SN}}$ as a function of $\phi_{1}$ and ratio $\phi_{3}/\phi_{1}$. SHM and optimal DHM ratios $\phi_{3}/\phi_{1}$ are shown as solid and dashed lines, respectively. (**c**) $\eta_{\mathrm{RIN}}$ as a function of $\phi_{1}$ for fixed ratios: $\phi_{3}/\phi_{1}=0.234$ for DHM; $\phi_{3}/\phi_{1}=0.288$ and $\phi_{5}/\phi_{1}=0.118$ for THM. (**d**) $\eta_{\mathrm{SN}}$ as a function of $\phi_{1}$ for fixed ratios: $\phi_{3}/\phi_{1}=0.188$ for DHM; $\phi_{3}/\phi_{1}=0.235$ and $\phi_{5}/\phi_{1}=0.080$ for THM. For comparison, the SWM case is shown as a function of 4ϕ/π in (**c**,**d**).

Theoretical calculations on ARW to obtain the DHM are shown in Figure 1a,b. To compare the performance of the DHM and THM with that of the SHM, we introduced improvement factors:

(16a) $$\eta_{\mathrm{RIN}}=\frac{{\mathrm{ARW}}_{\mathrm{RIN}}\left(\phi_{1},\phi_{3},\phi_{5}\right)}{{\mathrm{ARW}}_{\mathrm{RIN}}\left(2.70,0,0\right)}$$

(16b) $$\eta_{\mathrm{SN}}=\frac{{\mathrm{ARW}}_{\mathrm{SN}}\left(\phi_{1},\phi_{3},\phi_{5}\right)}{{\mathrm{ARW}}_{\mathrm{SN}}\left(2.70,0,0\right)},$$

which normalize the ARW to the optimal case for the SHM occurring at $\phi_{1}=2.70$. For ${\mathrm{ARW}}_{\mathrm{RIN}}$ in the DHM, the optimal modulation is $\left(\phi_{1},\phi_{3}\right)=\left(3.21,0.75\right)$, which results in a 2.3 dB improvement in the ARW. SN-induced ARW also improves by 0.7 dB at $\left(\phi_{1},\phi_{3}\right)=\left(3.18,0.60\right)$. For reference, we included results for negative ratios $-0.05<\phi_{3}/\phi_{1}<0$ in Figure 1a,b, which correspond to the case of a sinusoidal wave approximating a triangular wave. In general, negative ratios of $\phi_{3}/\phi_{1}$ deteriorate both ${\mathrm{ARW}}_{\mathrm{RIN}}$ and ${\mathrm{ARW}}_{\mathrm{SN}}$.

Figure 1c,d show the improvement factor dependence on the modulation index $\phi_{1}$ for the SHM, DHM, THM, and SWM. Here, we fixed the modulation index ratios for the DHM and THM to the value that minimize the ARW for each noise. One can see that incorporating more harmonics improves the ARW, and the improvement factor converges to that of the SWM. Optimal values of the SHM, DHM, and THM are summarized in Table 1.

According to the Fourier decomposition of a square wave in Equation (2), the modulation index ratios $\phi_{3}/\phi_{1}$ and $\phi_{5}/\phi_{1}$ that best approximate a modulation wave to a square wave are $\left(\phi_{3},\phi_{5}\right)/\phi_{1}$ = (1/3,1/5). Contrary to expectations, these ratios are not optimal for minimizing the ARW. It can be demonstrated through numerical calculations that optimal ratios will eventually converge to $\phi_{2n-1}/\phi_{1}=\frac{1}{2n-1}$ for $N_{h}\to \infty$, which corresponds to the SWM.

## 4. Experimental Setup

**Figure 2:**
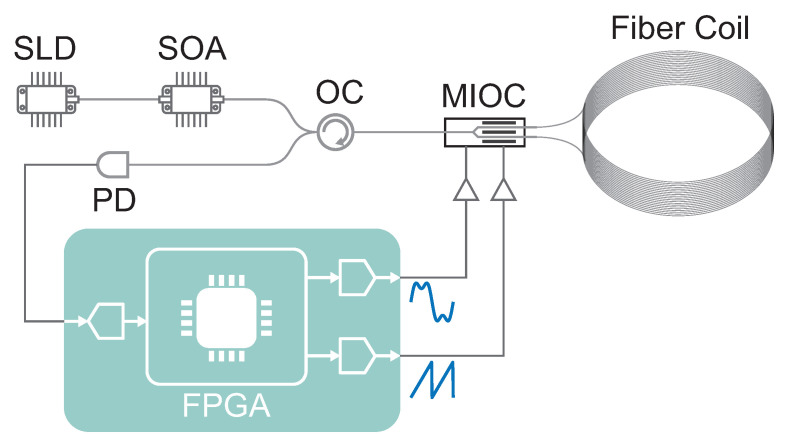
Schematic setup of the experimental apparatus. SLD, super-luminescent diode; SOA, semiconductor optical amplifier; OC, optical circulator; MIOC, multifunctional integrated optical chip; PD, photodetector.

The experimental setup is shown in Figure 2. The optical source is composed of a super-luminescent diode (SLD) with a central wavelength of 1544nm, bandwidth of 52nm, and output power of 40mW amplified by a semiconductor optical amplifier (SOA). The SOA has two important roles: it amplifies the output of the SLD to 100mW and reduces the RIN due to the gain saturation effect [23]. By comparing the noise measured directly after the SLD and SOA, we estimate a 11.2dB improvement in RIN. The output of the SOA goes through an optical circulator (OC) to a multifunctional integrated optical chip (MIOC). The output of the MIOC is spliced to a quadrupolar-wound coil having a length of L=4920m and an average radius of R=115mm. The return path of the optical circulator is inputted to a photodetector (PD), resulting in an output of $I_{0}=4\;\mathrm{mA}$. We positioned the fiber coil and MIOC inside a temperature-stabilized vacuum chamber (temperature $23.000\pm 0.001\;{}^{\circ }C$, pressure below $5\times 10^{-2}\;\mathrm{Pa}$) to reduce long-term drift effects.

The Sagnac phase detection system is a digital-closed-loop detection scheme built around a field-programmable gate array (FPGA) based on a Zynq 7020 SoC. Bias and serrodyne modulation signals are generated by a digital-to-analog converter (AD9747, 250 megasamples per second (MSPS)) followed by a homemade amplifier. Digital generation of the bias signal is crucially important for multi-harmonic modulation, as it ensures that the relative amplitude and phase between different harmonics remain constant. The output of the PD is read by an analog-to-digital converter (LTC2157-14, 250MSPS) and demodulated digitally using sinusoidal demodulation.

Calibration of the modulation index is realized by measuring the dependency between the demodulated signal $S_{1\omega }$ and the voltage applied to the MIOC and then fitting the resultant curve with a Bessel function (see Equation (6)). We employ the 101st harmonic of the coil eigenfrequency $f_{c}$ for modulation, which is given by $\omega /2\pi =101f_{c}=2.05\;\mathrm{MHz}$. At this frequency, the TPN is comparable to SN and DN. This will be analyzed in detail in the next section.

## 5. Experimental Results

We experimentally verify the improvement in the ARW by measuring its dependency on modulation index $\phi_{1}$ for the fixed ratios $\left(\phi_{3},\phi_{5}\right)/\phi_{1}$. The ARW including all sources of noise is given by:

(17) $$\mathrm{ARW}=\sqrt{{\mathrm{ARW}}_{\mathrm{TPN}}^{2}+{\mathrm{ARW}}_{\mathrm{RIN}}^{2}+{\mathrm{ARW}}_{\mathrm{SN}}^{2}+{\mathrm{ARW}}_{\mathrm{DN}}^{2}},$$

where the TPN-induced ARW is:

(18a) $${\mathrm{ARW}}_{\mathrm{TPN}}=\frac{\sigma_{\mathrm{TPN}}}{S_{F}I_{0}J_{1}^{1,3,5}\left(\phi_{1},\phi_{3},\phi_{5}\right)\sqrt{B}}$$

(18b) $$\sigma_{\mathrm{TPN}}=I_{0}\sqrt{\pi B\sum_{n=1}^{\infty }{\left[J_{2n-1}^{1,3,5}\left(\phi_{1},\phi_{3},\phi_{5}\right)-J_{2n+1}^{1,3,5}\left(\phi_{1},\phi_{3},\phi_{5}\right)\right]}^{2}\times \langle \Delta \phi_{N,\mathrm{rms}}^{2}\left(2n\omega \right)\rangle }.$$

Here,

(19) $$\begin{array}{cc} \langle \Delta \phi_{N,\mathrm{rms}}^{2}\left(2n\omega \right)\rangle =\frac{k_{B}T^{2}L}{\kappa \lambda^{2}}{\left(\frac{dn_{\mathrm{eff}}}{dT}+n_{\mathrm{eff}}\alpha_{L}\right)}^{2} & \\ & \times \ln\left[\frac{{\left(\frac{2}{W_{0}}\right)}^{4}+{\left(\frac{2n\omega }{D}\right)}^{2}}{{\left(\frac{4.81}{d}\right)}^{2}+{\left(\frac{2n\omega }{D}\right)}^{2}}\right]\left[1-\mathrm{sinc}\left(\frac{2n\omega Ln_{\mathrm{eff}}}{c}\right)\right] \end{array}$$

is the spectral density of phase noise introduced by the TPN. $k_{B}$, *T*, κ, $n_{\mathrm{eff}}$, $\alpha_{L}$, $W_{0}$ and *D* are the Boltzmann’s constant, the temperature of the coil, thermal conductivity, effective refractive index of the fiber, linear thermal expansion coefficient, mode field radius and thermal diffusivity, respectively. Typical values can be found in different studies [14,16,24].

The DN-induced ARW is given by:

(20a) $${\mathrm{ARW}}_{\mathrm{DN}}=\frac{\sigma_{\mathrm{DN}}}{S_{F}I_{0}J_{1}^{1,3,5}\left(\phi_{1},\phi_{3},\phi_{5}\right)\sqrt{B}}$$

(20b) $$\sigma_{\mathrm{DN}}=\sqrt{\frac{B}{2}}{\left\{2ei_{D}+i_{N}^{2}+{\left(\frac{v_{N}}{R_{F}}\right)}^{2}+\frac{4k_{B}T}{R_{F}}+{\left(\frac{v_{\mathrm{ADC}}}{R_{F}}\right)}^{2}\right\}}^{1/2},$$

where $i_{D}$, $i_{N}$, $v_{N}$, $R_{F}$ and $v_{\mathrm{ADC}}$ are the dark current of the PD, the input noise current and voltage of the transimpedance amplifier, the feedback resistance, and the input noise voltage of the ADC, respectively.

**Figure 3:**
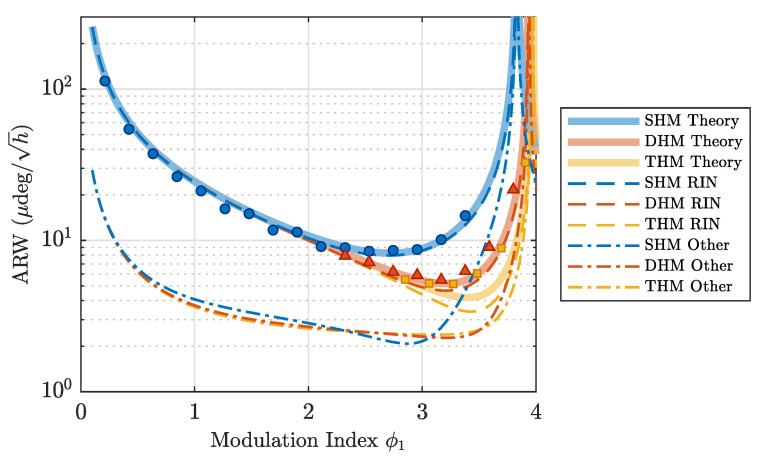
ARW dependence on the modulation index $\phi_{1}$ for SHM, DHM ($\phi_{3}/\phi_{1}=0.234$) and THM ($\left(\phi_{3},\phi_{5}\right)/\phi_{1}=\left(0.288,0.118\right)$) denoted by circular, triangular and square points, respectively. Solid lines: Total estimated ARW including all sources of noise. Dashed lines: RIN-induced ARW. Dashed-dotted lines: SN-, DN- and TPN-induced ARW.

The results are shown in Figure 3. We utilize the fixed ratios $\left(\phi_{3},\phi_{5}\right)/\phi_{1}=\left(0.234,0\right)$ for DHM and $\left(\phi_{3},\phi_{5}\right)/\phi_{1}=\left(0.288,0.118\right)$ for THM, corresponding to the optimal ratios that minimize the RIN effects. Each value was estimated by fitting the Allan deviation during one hour of measurement. The best ARW values obtained for SHM, DHM and THM were $8.5\left(2\right)\;\mu \deg/\sqrt{h}$, $5.5\left(4\right)\;\mu \deg/\sqrt{h}$ and $5.2\left(2\right)\;\mu \deg/\sqrt{h}$, respectively, which translates to a 1.9dB improvement for DHM and 2.2dB for THM. Solid lines indicate the theoretical estimation for ARW including all sources of noise (Equation (17)).

We estimated the RIN (dashed lines in Figure 3) by switching off the modulation and measuring the noise in the detector in open-loop mode. Since RIN is one order of magnitude larger than SN, the resultant noise is dominated by RIN. The estimated attenuation factor was η=0.15. Detection noise was estimated in the same manner with the input light in the PD removed. The measured DN was 3% of RIN in the absence of modulation. Other noise-induced ARWs, shown in dashed-dotted lines in Figure 3, include the effects of SN, DN and TPN.

**Figure 4:**
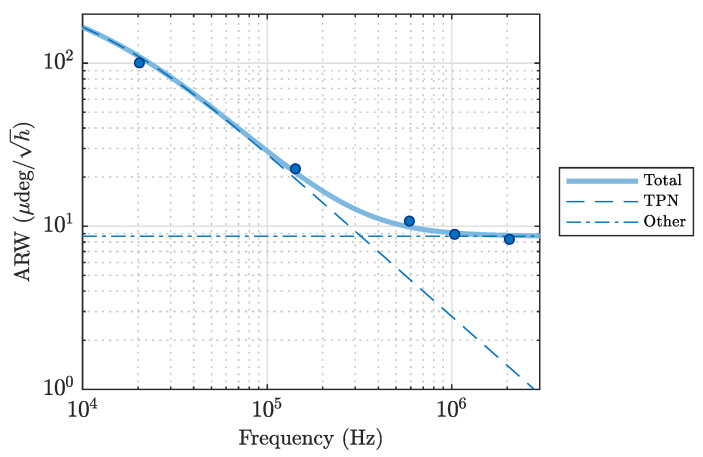
Modulation frequency dependency of the ARW for the SHM case. Solid dots: Experimental results. Solid line: ARW including all sources of noise. Dashed line: TPN contribution to ARW. Dashed-dotted lines: Sum of other noise-induced ARW, including SN, DN and RIN.

To estimate the amount of TPN, we measured the dependency of the ARW on the modulation frequency for the SHM case (modulation index $\phi_{1}=2.70$). The experimental results are shown in Figure 4. Since the other sources of noise can be considered independent of the modulation frequency, the data can be fitted by:

(21) $$\mathrm{ARW}=\sqrt{{\mathrm{ARW}}_{\mathrm{TPN}}^{2}\left(\omega ;D\right)+{\mathrm{ARW}}_{\mathrm{other}}^{2}}$$

where the fitting parameters are the thermal diffusivity of the optical fiber *D* and a constant ${\mathrm{ARW}}_{\mathrm{other}}$ that includes RIN, DN and SN contributions to the ARW. This resulted in $D=1.0\left(1\right)\times 10^{-6}\;m^{2}s^{-1}$ and ${\mathrm{ARW}}_{\mathrm{other}}=8.7\left(8\right)\;\mu \deg/\sqrt{h}$. We found no discrepancy between the obtained thermal diffusivity value and the value for bulk fused silica found in the literature [14,16,28].

The estimated contributions of RIN, DN, SN and TPN to ARW are summarized in Table 2. For a modulation frequency of 2.05MHz, the TPN contribution to the ARW is comparable to the sum of the DN and SN contributions. Consequently, a further increase in the modulation frequency will result in a negligible improvement in the ARW. On the other hand, RIN is still the dominant source of noise. In principle, increasing the number of harmonics in the modulation to $N_{h}=4$ or more can decrease the RIN. However, we found that even THM produced very little improvement in ARW. The reason for this is still unclear. One possible explanation is that the high frequency of the fifth harmonic (5ω/2π=10MHz) produces nonlinearity effects in the electronics and optoelectronics that deteriorate the performance of THM. Another possibility is that environmental vibrations are limiting the ARW. We observed that our system is highly susceptible to building vibrations caused by human activity and wind. Regarding SHM and DHM, we found good agreement between the theory and experiments.

**Figure 5:**
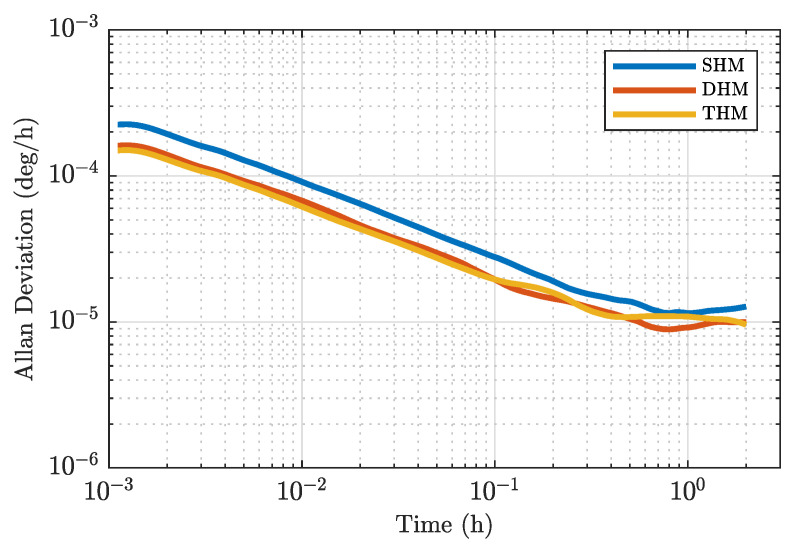
Allan deviation for 12-hour measurements of SHM, DHM, and THM.

The long-term performance of our FOGs is shown in Figure 5. The obtained ARWs for SHM, DHM and THM were $9.0\left(1\right)\;\mu \deg/\sqrt{h}$, $6.5\left(2\right)\;\mu \deg/\sqrt{h}$, and $6.1\left(1\right)\;\mu \deg/\sqrt{h}$, respectively. The bias instability was ∼10μdeg/h for the three cases. We observed no degradation of long-time stability due to multiple-harmonic modulation.

## 6. Comparison with Square-Wave Modulation

**Figure 6:**
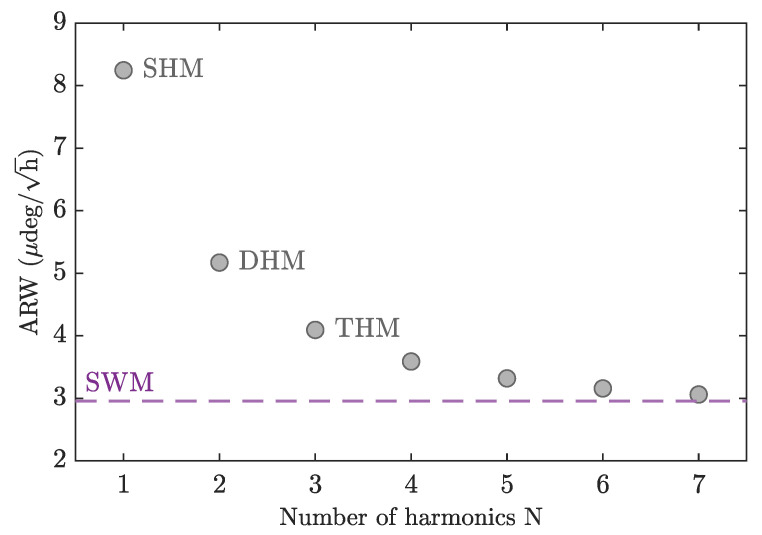
Dependence of the ARW on the number of modulation harmonics. The dashed line shows the best ARW attainable using ideal square-wave modulation.

When a high frequency is used for bias modulation, the spike problem hampers the application of SWM. However, from a theoretical point of view, it is useful to compare the performance of multi-harmonic modulation with ideal, spike-free SWM to estimate the number of required harmonics. Figure 6 shows the dependence of the ARW on the number of harmonics. For each number of harmonics, we calculate the optimal set of modulation indexes $\phi_{i}$ that minimize the ARW when the effects of all types of noise (RIN, TPN, SN, and DN) are considered using the parameters of our current experimental setup. With three harmonics, corresponding to THM, the ARW is reduced by less than half compared with the case of one harmonic. Further increasing the number of harmonics further improves the ARW, but very slowly. Ultimately, the ARW converges to the optimal ${\mathrm{ARW}}_{\mathrm{SWM}}$ that can be obtained with ideal SWM, shown by the dashed line in Figure 6. The optimal modulation index for SWM is estimated as ϕ=2.81, which results in ${\mathrm{ARW}}_{\mathrm{SWM}}=2.9\;\mu \deg/\sqrt{h}$. The contributions to ${\mathrm{ARW}}_{\mathrm{SWM}}$ from RIN, DN, TPN and SN are $1.7\;\mu \deg/\sqrt{h}$, $1.7\;\mu \deg/\sqrt{h}$, $1.3\;\mu \deg/\sqrt{h}$, and $1.1\;\mu \deg/\sqrt{h}$, respectively.

Note that this analysis is not universal but rather depends on the amount of noise in each individual setup. This is because SWM is very effective at reducing the effects of RIN and SN but offers no advantage in reducing the effects of TPN and DN compared with sinusoidal modulation. For a system with a large amount of TPN and/or DN, one or two harmonics will be sufficient to reach a value close to ${\mathrm{ARW}}_{\mathrm{SWM}}$. On the other hand, for a system with a large amount of RIN, more harmonics will be required.

## 7. Conclusions

In this study, we have introduced a novel modulation method using multiple harmonics of sinusoidal modulation. This method combines the advantages of SWM and sinusoidal modulation to simultaneously suppress TPN and RIN effects. In Section 2, we derived the theoretical formulas used to calculate the noise and signal levels for DHM and THM. In Section 3, we determined the optimal parameters for modulation. We found that the RIN-induced ARW can be reduced by 2.3 dB and 3.7 dB in the DHM and THM cases, respectively. Additionally, the SN-induced ARW is reduced by 1.0 dB in the THM case. Furthermore, in Section 1, we experimentally demonstrated improvement of the ARW using multi-harmonic modulation. The ARW was improved from $8.5\;\mu \deg/\sqrt{h}$ in SHM to $5.5\;\mu \deg/\sqrt{h}$ and $5.2\;\mu \deg/\sqrt{h}$ in the DHM and THM cases, respectively. Finally, in Section 6, we compared the performance of multi-harmonic modulation with the ideal SWM case.

Table 3 shows a comparison between the FOG presented in this work and other high-accuracy FOGs. The phase noise in our FOG is notably smaller than that in other works by virtue of the simultaneous reduction in TPN and RIN achieved using a high modulation frequency combined with multi-harmonic modulation. This simultaneous reduction would not be possible using SWM due to the spike problem, which prevents its use at high modulation frequencies. On the other hand, sinusoidal modulation is compatible with the high modulation frequencies necessary to reduce TPN, but it performs poorly in reducing RIN.

An inertial navigation system (INS) with an accuracy of one nautical mile per month requires a gyro bias below 20μdeg/h [29]. For initial alignment purposes, reaching this bias precision within one hour requires an ARW below $20\;\mu \deg/\sqrt{h}$ [29]. Our system, using either DHM or THM, can reach a minimum bias stability of 10μdeg/h in less than one hour. With the phase noise reported here, it is possible to reduce the radius of our coil to R=30mm while maintaining an ARW of $20\;\mu \deg/\sqrt{h}$, paving the way for the production of a compact INS with an accuracy of one nautical mile in a month.

**Table 3:** Comparison of high-accuracy FOGs.

Modulation Method	Phase Noise	Scale Factor	ARW	Ref.
Square-wave	$0.68\;\mu \mathrm{rad}/\sqrt{\mathrm{Hz}}$	34s	$69\;\mu \deg/\sqrt{h}$	[30]
Sinusoidal	$0.55\;\mu \mathrm{rad}/\sqrt{\mathrm{Hz}}$	157s	$12\;\mu \deg/\sqrt{h}$	[16]
Square-wave	$0.13\;\mu \mathrm{rad}/\sqrt{\mathrm{Hz}}$	12s	$37\;\mu \deg/\sqrt{h}$	[4] ^1^
Sinusoidal	$68\;\mathrm{nrad}/\sqrt{\mathrm{Hz}}$	15.5s	$15\;\mu \deg/\sqrt{h}$	[24]
Multi-harmonic	$23\;\mathrm{nrad}/\sqrt{\mathrm{Hz}}$	15.5s	$5.2\;\mu \deg/\sqrt{h}$	This Work

^1^ 1st mockup.

To further reduce RIN and TPN, modulation with even higher frequencies and number of harmonics is needed. Ultimately, the bandwidth and linearity of the electronic and optoelectronic components will limit how much RIN and TPN can be decreased using this method. Improving these technologies will be important in the future to increase the precision of FOGs.

This work has focused on the improvement of the ARW using multi-harmonic modulation. Long-term stability of an FOG is crucial to improve the accuracy of an INS. Although we observed no degradation in bias instability due to DHM or THM, future research will be required to improve the long-term stability.

## Figures and Tables

**Table 1:** Optimal modulation indexes for SHM, DHM and THM that minimize ${\mathrm{ARW}}_{\mathrm{RIN}}$ and ${\mathrm{ARW}}_{\mathrm{SN}}$. Improvement factors are shown in dB units.

	RIN				Shot Noise			
	$\phi_{1}$	$\phi_{3}$	$\phi_{5}$	$\eta_{\mathrm{RIN}}$	$\phi_{1}$	$\phi_{3}$	$\phi_{5}$	$\eta_{\mathrm{SN}}$
**SHM**	2.70	0	0	0	2.70	0	0	0
**DHM**	3.21	0.75	0	−2.3	3.18	0.60	0	−0.7
**THM**	3.42	0.99	0.41	−3.7	3.40	0.80	0.27	−1.0

**Table 2:** Estimation of the contribution for each type of noise to the total ARW.

	Modulation Index			ARW ($\mu \deg/\sqrt{h}$)					
	$\phi_{1}$	$\phi_{3}/\phi_{1}$	$\phi_{5}/\phi_{1}$	**RIN**	**SN**	**TPN**	**DN**	**Total**	**Experiment**
**SHM**	2.53	0	0	8.09	1.41	1.68	0.74	8.42	8.5(2)
**DHM**	3.17	0.234	0	4.67	1.21	1.62	1.03	5.19	5.5(4)
**THM**	3.27	0.288	0.118	3.56	1.17	1.78	1.10	4.30	5.2(2)

## Data Availability

Not applicable.

## Abbreviations

The following abbreviations are used in this manuscript:

FOG	Fiber-optic gyroscope
RIN	Relative-intensity noise
TPN	Thermal-phase noise
SN	Shot noise
DN	Detection noise
SWM	Square-wave modulation
SHM	Single-harmonic modulation
DHM	Double-harmonic modulation
THM	Triple-harmonic modulation
OBF	Ordinary Bessel function
GBF	Generalized Bessel function
ARW	Angular random walk
PD	Photodetector
SLD	Super-luminescent diode
SOA	Semiconductor optical amplifier
OC	Optical circulator
MIOC	Multifunctional integrated optical chip
FPGA	Field-programmable gate array
MSPS	Megasamples per second
